# A physiologically based pharmacokinetic (PBPK) model to describe organ distribution of ^68^Ga-DOTATATE in patients without neuroendocrine tumors

**DOI:** 10.1186/s13550-021-00821-7

**Published:** 2021-08-16

**Authors:** H. Siebinga, B. J. de Wit-van der Veen, J. H. Beijnen, M. P. M. Stokkel, T. P. C. Dorlo, A. D. R. Huitema, J. J. M. A. Hendrikx

**Affiliations:** 1 Department of Pharmacy and Pharmacology, The Netherlands Cancer Institute, Amsterdam, The Netherlands; 2 Department of Nuclear Medicine, The Netherlands Cancer Institute, Amsterdam, The Netherlands; 3 Department of Clinical Pharmacy, University Medical Center Utrecht, Utrecht University, Utrecht, The Netherlands; 4 Department of Pharmacology, Princess Máxima Center for Pediatric Oncology, Utrecht, The Netherlands

**Keywords:** PBPK modeling, Whole-body distribution, ^68^Ga-DOTATATE, PRRT, Peptide amount, SSTR2

## Abstract

**Background:**

Physiologically based pharmacokinetic (PBPK) models combine drug-specific information with prior knowledge on the physiology and biology at the organism level. Whole-body PBPK models contain an explicit representation of the organs and tissue and are a tool to predict pharmacokinetic behavior of drugs*.* The aim of this study was to develop a PBPK model to describe organ distribution of ^68^Ga-DOTATATE in a population of patients without detectable neuroendocrine tumors (NETs).

**Methods:**

Clinical ^68^Ga-DOTATATE PET/CT data from 41 patients without any detectable somatostatin receptor (SSTR) overexpressing tumors were included. Scans were performed at 45 min (range 30–60 min) after intravenous bolus injection of ^68^Ga-DOTATATE. Organ (spleen, liver, thyroid) and blood activity levels were derived from PET scans, and corresponding DOTATATE concentrations were calculated. A whole-body PBPK model was developed, including an internalization reaction, receptor recycling, enzymatic reaction for intracellular degradation and renal clearance. SSTR2 expression was added for several organs. Input parameters were fixed or estimated using a built-in Monte Carlo algorithm for parameter identification.

**Results:**

^68^Ga-DOTATATE was administered with a median peptide amount of 12.3 µg (range 8.05–16.9 µg) labeled with 92.7 MBq (range 43.4–129.9 MBq). SSTR2 amounts for spleen, liver and thyroid were estimated at 4.40, 7.80 and 0.0108 nmol, respectively. Variability in observed organ concentrations was best described by variability in SSTR2 expression and differences in administered peptide amounts.

**Conclusions:**

To conclude, biodistribution of ^68^Ga-DOTATATE was described with a whole-body PBPK model, where tissue distribution was mainly determined by variability in SSTR2 organ expression and differences in administered peptide amounts.

## Introduction

Neuroendocrine tumors (NETs) are a heterogeneous family of malignancies that arise from neuroendocrine cells and are often expressed in the gastroenteropancreatic tract and the bronchopulmonary tree [1, 2]. NETs show an overexpression of somatostatin receptors (SSTRs), which offers the possibility for imaging and treatment with radionuclide-labeled somatostatin analogues [3, 4]. This is nowadays referred to as a theranostic approach, using, for example, gallium-68-labeled DOTA–D-Phe^1^-Tyr^3^-Thr^8^-octreotate (^68^Ga-DOTATATE) PET/CT for lesion localization, pre-treatment patient selection and post-treatment follow-up and lutetium-177 (^177^Lu)-labeled DOTATATE for peptide receptor radionuclide therapy (PRRT) [5, 6]. The clinical application of this specific approach in NETs has strengthened since the publication of the results of the phase-III NETTER-1 trial [7].

For ^68^Ga-DOTATATE, several clinical studies have already been performed regarding aspects such as dosimetry, optimal imaging parameters and diagnostic value [8–11]. However, controversies remain on the value of ^68^Ga-DOTATATE accumulation to predict response to PRRT and hence its role in patient selection [12, 13]. Although discrepancies between ^68^Ga- and ^177^Lu-DOTATATE accumulation in clinical studies are often explained by their retrospective nature and small patient series, this may also rely on actual differences between the ligands in pharmacokinetic (PK) behavior and receptor interactions. Detailed knowledge on these aspects for both ^68^Ga- and ^177^Lu-DOTATATE is currently lacking, making interpretation of these complex interactions and subsequent optimization of clinical algorithms difficult. Yet, recent small-scale studies do suggest that optimization of radiopharmaceutical amount, administered activity and fractionation schemes can lead to higher individual response rates and lower toxicity profiles [14–18].

One method to gain a more mechanistic understanding of PK behavior and biodistribution of drugs is physiologically based pharmacokinetic (PBPK) modeling. Results of such in silico models are widely used in the pharmaceutical industry (mainly non-radiopharmaceuticals) for study design, dose selection of new compounds of interest and improvement in the use of European Medicines Agency (EMA)- and/or Food and Drug Administration (FDA)-approved drugs [19, 20].

PBPK models combine drug-specific information with independent prior knowledge on the physiology and biology at the organism level (i.e., system-specific information). The combination of this information eventually leads to a mechanistic representation of the behavior of the drug in an organism, so that drug concentration–time profiles can be predicted a priori [21–23]. Whole-body PBPK models contain an explicit representation of organs and tissues that have a relevant impact on the absorption, distribution, metabolism and excretion (ADME) of the drug*.* These whole-body models are currently widely used as it is possible to simulate concentration–time profiles for each specific organ and thus distribution throughout the whole body. When these concentration–time profiles are converted into time-radioactivity profiles and coupled to radionuclide characteristics, predictions can be made on average absorbed dose (Gray, Gy) to target tissues or organs.

Several PBPK models for radiolabeled somatostatin analogues have already been published, for instance, to assess the effect of tumor volume on whole-body distribution, or to evaluate the impact of different peptide amounts [14–17, 24–26]. These models were all developed to increase the so-called therapeutic indices (i.e., ratio between tumor and normal-organ dose) and hence included data from NET patients with relatively high tumor burdens. In contrast, the PBPK model presented here will specifically describe the distribution of radiolabeled DOTATATE in normal organs alone. To gain an unbiased mechanistic understanding of PK behavior and biodistribution, identification of the most relevant processes that influence drug ADME is key. Whole-body accumulation of radiolabeled DOTATATE is potentially affected by tumor burden, meaning that large tumor volumes with receptor overexpression can decrease uptake in both reference organs such as the liver and spleen and organs-at-risk like the kidneys. By eliminating this prominent aspect of tumor-related distribution, other relevant factors may be identified more accurately. In addition, development of a PBPK model using different software tools and observed clinical data is an extra verification of the previously developed ^68^Ga-DOTATATE models [14, 26]. Also, the use of an open-source software tool might make PBPK models even more accessible and convenient to a larger public. Lastly, the developed PBPK models lack a great number of clinical observations to validate and thus predict whole-body distribution for a larger population.

Therefore, the aim of this study was to develop a PBPK model to describe normal organ distribution of ^68^Ga-DOTATATE for a population of patients without NETs. In future, this ‘normal-organ’ PBPK model can be extended by implementing tumor volumes, ^177^Lu-radiolabeled DOTATATE and, subsequently, coupling concentration–time profiles to clinical outcomes. Based on this work, we hope to better identify discrepancies between ^68^Ga- and ^177^Lu-DOTATATE and hence improve the therapeutic indices and patient selection based on ^68^Ga-DOTATATE PET/CT.

## Methods

### Patient data

The study was approved by the institutional review board (IRB) of the Netherlands Cancer Institute in Amsterdam, the Netherlands (IRBd18078), and only data were used of patients who had given consent via institutional procedures. All available clinical ^68^Ga-DOTATATE PET/CT data from patients without any clinical evidence of active disease nor SSTR overexpressing NETs on PET/CT (defined as no increased uptake above local tissue background) acquired between August 2011 and April 2016 were selected (*n* = 41). Organ accumulation of ^68^Ga-DOTATATE measured on these PET/CT scans was used to evaluate the developed PBPK model.

### Scan protocol

Somatostatin analogue therapy was withdrawn prior to ^68^Ga-DOTATATE administration. Scans were performed according to local clinical protocol at 45 min (range 30–60 min) after intravenous bolus injection of approximately 100 MBq ^68^Ga-DOTA–D-Phe^1^-Tyr^3^-Thr^8^-octreotate (~ 10 µg total peptide). ^68^Ga-DOTATATE was prepared according to locally validated procedures and national legislation on radiopharmaceuticals. The ^68^Ga concentrations (Bq/mL) were quantified on PET scans obtained on a Gemini ToF PET/CT (Philips, the Netherlands) with 2–2.5 min per bed position. In addition, a low-dose CT was acquired for attenuation correction and anatomical correlation. Organ (spleen, liver and thyroid) and blood (aorta) radioactivity concentrations were determined non-invasively from circular volumes of interest (VOIs) with a diameter of at least 20 mm drawn over the organ or aorta to identify the mean activity levels. After a decay correction to injection time, corresponding peptide concentrations (µg/L) per organ were calculated based on apparent specific activities (MBq/µg) available in the production documents of each labeling. The calculated total administered DOTATATE peptide (bound and unbound) was used as input of the administered dose for the model; therefore, no further decay correction for ^68^Ga in the model was performed.

### PBPK model development

A whole-body PBPK model was developed using the protein base model in PK-Sim and MoBi (Open Systems Pharmacology Suite, version 8.0) [27]. This software enables access to all relevant anatomical and physiological parameters for humans. Data such as reference organ volumes, organ densities, blood flows, blood volumes and renal function have already been incorporated based on the relevant literature [28]. Specific physicochemical information about ^68^Ga-DOTATATE and relevant biological processes linked to its in vivo behavior was manually added to the PBPK model in order to eventually describe concentration–time profiles of the drug.

The following key parameters were implemented to create the in-house developed PBPK model of ^68^Ga-DOTATATE. Compound-specific physicochemical parameters that could be defined based on the previous literature were molecular weight, lipophilicity, fraction unbound in plasma and p*K*_a_ values. There is limited knowledge about the metabolism and excretion of ^68^Ga-DOTATATE, although it is known that 12% of the administered dose is excreted unchanged in the urine within the first 4 h [9]. Therefore, renal clearance was added to the model as a specific excretion route and was manually scaled to a predicted 12% unchanged excretion in urine. ^177^Lu-DOTATATE does not undergo hepatic clearance; therefore, the same is expected for ^68^Ga-DOTATATE [29]. For this reason, hepatic clearance was not added to the model.

All organs were automatically included in the human model by the software, and each organ compartment was subdivided into vascular, interstitial and intracellular compartments. Distribution within these organ compartments was assumed to be homogenous. Also, the thyroid gland was added to the standardized organism using previously published data [30]. The organs were linked by arterial and venous blood compartments, and each organ was further characterized by a specific blood flow, volume, tissue-partition coefficient and permeability [21, 22]. Individual-specific input parameters (such as height, body weight and age) were based on the medians of the population data that were used for validation.

DOTATATE binds to the SSTRs on the cell membrane of organs and tumors, whereafter the complex is internalized into the cells [31, 32]. To describe this physiological ^68^Ga-DOTATATE target accumulation, SSTR2 was added to the membrane surface of all organs that are known to express this receptor [9, 33]. Other SSTRs and their expression profiles were neglected, because of their limited effect on overall peptide disposition due to low affinity or low expression [34–36].

Passage of ^68^Ga-DOTATATE into the intracellular compartment was only made possible by internalization of SSTR2 into the cell after binding of the peptide to the receptor. After this internalization, SSTR2 and the radiopharmaceutical dissociate intracellularly, followed by rapid recycling of the receptor back to the cell membrane [24, 32, 37]. Receptor recycling was added as a zero-order kinetic reaction to model. ^68^Ga-DOTATATE was assumed to remain intracellularly after internalization, based on evaluation of clinical PET/CT scans, but also because passive diffusion is unlikely with its high molecular weight. Intracellular ^68^Ga-DOTATATE degradation was added into the model as an unknown first-order reaction. For reasons of model simplicity, a fixed degradation constant was added to all compartments and no degradation products were included in the model [16]. The internalization reaction was based on a previously published PBPK model for ^90^Y-DOTATATE including SSTR2 receptors [16] and consisted of separate reactions for peptide receptor binding (nonlinear) and total internalized peptide amount. These two reactions are described as follows (with “*i*” referring to a corresponding organ):

1 $$\frac{{{d}}}{{{{d}}t}}{{Complex}}_{i} = \frac{{k_{{{{off}}}} }}{{K_{{{D}}} }}*R_{i} *P_{i} *K_{{{{water}},i}} - k_{{{{off}}}} *{{Complex}}_{i}$$

where *k*_*off*_ is the dissociation rate constant of ^68^Ga-DOTATATE from the SSTR receptor (min^−1^), *K*_*D*_ is the dissociation constant (nmol/L), *R*_*i*_ is the SSTR2 receptor expression in the specific organ (nmol), *P*_*i*_ is the interstitial peptide concentration of DOTATATE (nmol/L), *K*_*water,i*_ is the partition coefficient (water/compartment) and *Complex*_*i*_ is the amount of SSTR2 occupied with ^68^Ga-DOTATATE (nmol).

2 $$\frac{{\text{d}}}{{{\text{d}}t}}P_{{{\text{intracellular}}, i }} = k_{{{\text{int}}}} *{\text{Complex}}_{i} - k_{{{\text{deg}}}} *P_{i}$$

where *k*_*int*_ is the internalization rate constant (min^−1^), *Complex*_*i*_ is the amount of SSTR2 bound to ^68^Ga-DOTATATE (nmol), *k*_*deg*_ is the degradation rate constant (min^−1^) and* P*_*i*_ is the intracellular peptide amount of ^68^Ga-DOTATATE (nmol).

### Model fitting and verification

Input parameters were fixed or fitted based on prior knowledge of these parameters. For model evaluation, the concentration for SSTR2 in the interstitial compartment of the organ was estimated for spleen, liver and thyroid. Model fitting was performed using a built-in Monte Carlo algorithm for parameter identification to optimize selected input parameter to describe the data best. The total model fit was evaluated based on a residual sum of squares (total error). Range for parameter fitting was 0–250 nmol/L for SSTR2 reference concentration (similar to spleen SSTR2 concentration). The SSTR2 amounts were fitted to the clinical data (peptide concentrations (µg/L)) of all 41 patients combined, and these observed scan data were assigned to whole-organ predictions including vascular, interstitial and intracellular compartments of that specific organ. All data points for the spleen, liver and thyroid were used during the model fitting of the SSTR2 concentrations. This resulted in one prediction for each organ representing this population. Ranges of SSTR2 concentrations per organ were obtained by scaling predictions to minimum and maximum observed values while also taking into account differences in administered peptide amount. This resulted in estimated population minimum and maximum SSTR2 densities per organ.

In addition, a sensitivity analysis was completed in MoBi to calculate the sensitivity of the PK model output, which was performed by alteration of input parameters with ± 10% [38]. All input parameters were evaluated using the sensitivity analysis, and this provided understanding of critical input parameters for model output and thus predictions. The sensitivity (*S*_*i,j*_) was calculated using the following equation:

3 $$S_{i,j} = \frac{{\Delta {{PK}}_{j} }}{{\Delta p_{i} }}*\frac{{p_{j} }}{{{{PK}}_{j} }}$$

where *PK*_*j*_ is the PK parameter of a certain output to an input parameter (*p*_*j*_). Thus, the sensitivity for the PK parameter to that input parameter was calculated as the ratio of the relative change of that PK parameter (Δ*PK*_*j*_) and the relative variation of the input parameter (Δ*p*_*i*_). A sensitivity value of -1 implies that a 10% increase of the input parameter resulted in a 10% decrease of the PK parameter output.

### Statistical test

A Pearson’s correlation coefficient test was performed in R (version 3.6.3), to investigate a potential correlation between administered DOTATATE amount and observed organ uptake [39]. A p-value less than 0.05 was considered statistically significant.

## Results

Organ accumulation of ^68^Ga-DOTATATE measured on PET/CT scans of 41 patients was included in this study to evaluate an in-house developed PBPK model for ^68^Ga-DOTATATE. Administered peptide content varied along the population with a median administered DOTATATE amount of 12.3 µg (range 8.05–16.9 µg). Injected radioactivity labeled to the peptide was 92.7 MBq (range 43.4–129.9 MBq). Patient characteristics and demographics are listed in Table 1.

**Table 1:** Patient characteristics and demographics

Characteristics	Median (range), mean ± SD or *n* (%)
Sex	
Males	22 (53.7%)
Females	19 (46.3%)
Age (years)	58 (22–79)
Body weight (kg)	76 (53–120)
Height (cm)	174 (155–196)
Renal function (eGFR; mL/min/1.73m^2^)	77.5 (61.0–122)
Scan time after injection (min)	47 (35–78)
Administered peptide amount (µg)	12.3 (8.05–16.9)
Administered radioactivity (MBq)	92.7 (43.4–130)
SUV_max_	
Aorta	1.59 ± 0.45
Spleen	21.0 ± 4.50
Liver	8.52 ± 2.11
Thyroid	4.42 ± 1.53
SUV_peak_	
Aorta	1.17 ± 0.36
Spleen	19.0 ± 4.19
Liver	7.16 ± 1.58
Thyroid	3.20 ± 1.24

SUV: standardized uptake value

**Fig. 1:**
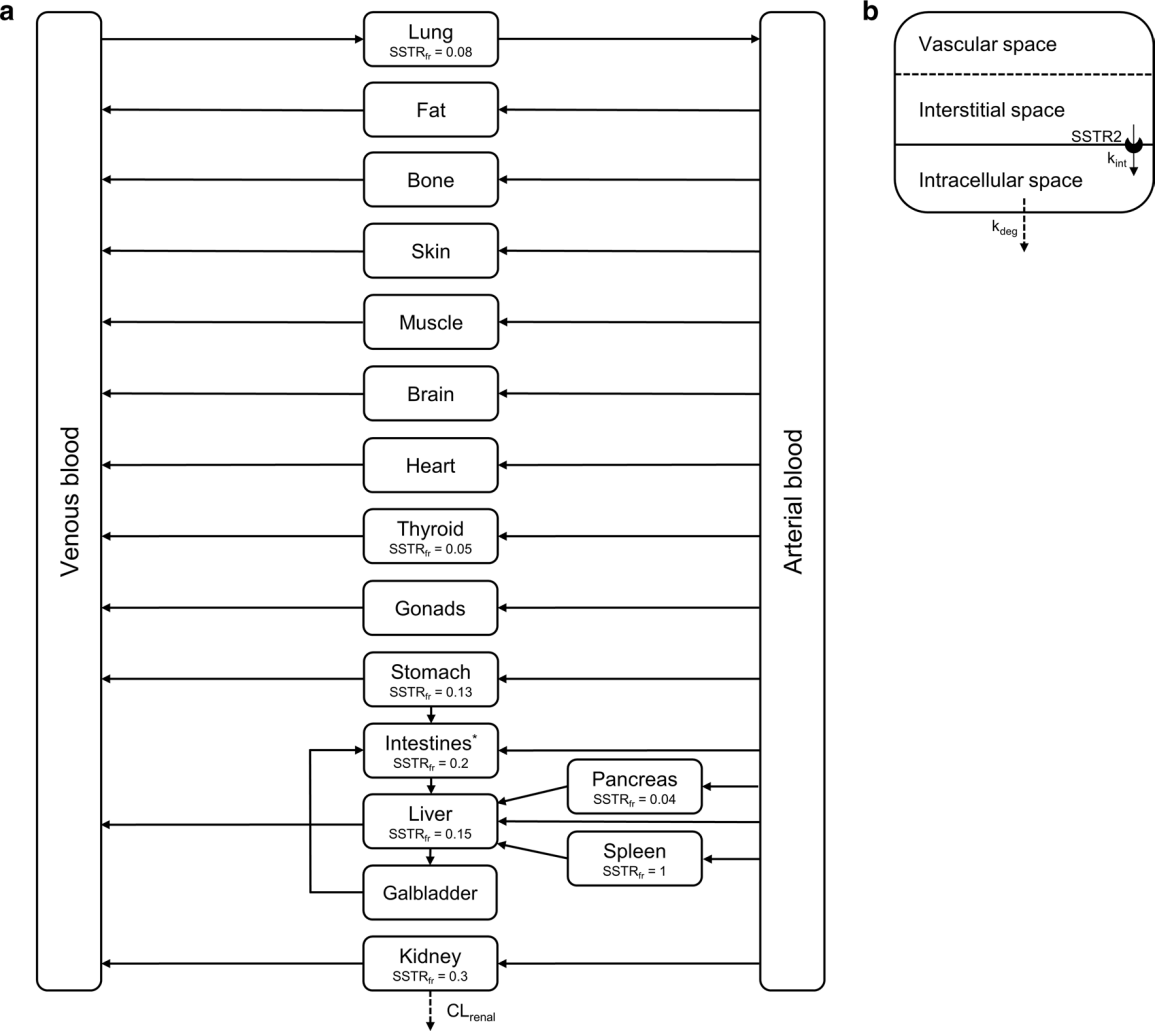
Schematic overview of the developed ^68^Ga-DOTATATE PBPK model (**a**), with all organ compartments included and arrows representing blood flow. Each organ compartment consists of subcompartments (**b**), where peptide transfer from interstitial to intracellular space occurs by internalization of the SSTR2 receptor after peptide binding. *Intestinal compartment consists of small intestine (SSTR2 fraction = 0.09) and large intestine (SSTR2 fraction = 0.11). CL_renal_: renal clearance; *k*_int_: internalization rate; *k*_deg_: degradation rate; SSTR_fr_: somatostatin receptor fraction

Based on the previous literature, molecular weight, lipophilicity and p*K*_*a*_ values used were 1502.3 g/mol, − 3.69 and 0.46 (strongest acidic) and 10.3 (strongest basic), respectively [40, 41]. Plasma protein binding for ^68^Ga-DOTATATE was fixed to the reported value of 0.31 [42]. The dissociation constant (*K*_D_) and dissociation rate constant (*k*_off_) were fixed to 0.20 nmol/L and 0.012 min^−1^, respectively. These values were based on the measured half maximal inhibitory concentration (IC_50_) by Reubi et al. and the association rate constant (*k*_on_) of 1 × 10^6^ L/mol/s [43]. The calculated *k*_off_ value was in accordance with previously reported values for similar peptides [15, 16, 24, 44]. Internalization rates were assumed to be constant over time and fixed to 0.161 min^−1^ for all organs, based on the internalization half-life of somatostatin-14 (SS14) [45]. The SSTR2 recycling rate was fixed to 0.059 nmol/min, which was calculated based on the receptor recycling half-life of SS14 and a start amount of 5 nmol [45]. The degradation rate constant of 0.00012 min^−1^ was based on previously published NET PBPK models with explicitly modeled SSTR2 expression [16, 17, 24]. All fixed and initial input parameters and results for the estimated parameters are shown in Table 2. Spleen has the highest SSTR2 organ density and, thus, was modeled as fraction 1 and was set as the SSTR2 reference concentration. Other organ fractions were initially based on the literature and then fitted to the observed clinical data during model verification [9, 33, 46]. An overview of the whole-body PBPK model with initial SSTR2 input fractions is provided in Fig. 1.

**Table 2:** All initial, fixed and fitted input parameters for the whole-body PBPK model of ^68^Ga-DOTATATE

Parameter	Initial or fixed value	Fitted value	References
Molecular weight	1502.3 g/mol		[40, 41]
Lipophilicity	− 3.69		[41]
p*K*_*a*_ (strongest acidic)	0.46		[40]
p*K*_*a*_ (strongest basic)	10.3		[40]
*K*_D_	0.20 nmol/L		[24, 43]
*k*_off_	0.012 min^−1^		[24, 43]
*k*_int_	0.161 min^−1^		[45]
*k*_deg_	0.00012 min^−1^		[16]
Fraction unbound	0.69		[42]
Interstitial SSTR2 concentration spleen	100 nmol/L	112.0 nmol/L	[8, 16]
Interstitial SSTR2 concentration liver	15 nmol/L	20.2 nmol/L	[8, 16]
Interstitial SSTR2 concentration thyroid	5.0 nmol/L	4.71 nmol/L	[8]
Renal plasma clearance	0.45 ml/min/kg	0.67 ml/min/kg	[29]

*K*_D_: equilibrium dissociation constant; *k*_off_: dissociation rate constant; *k*_int_: internalization rate; *k*_deg_: degradation rate; SSTR: somatostatin receptor

**Fig. 2:**
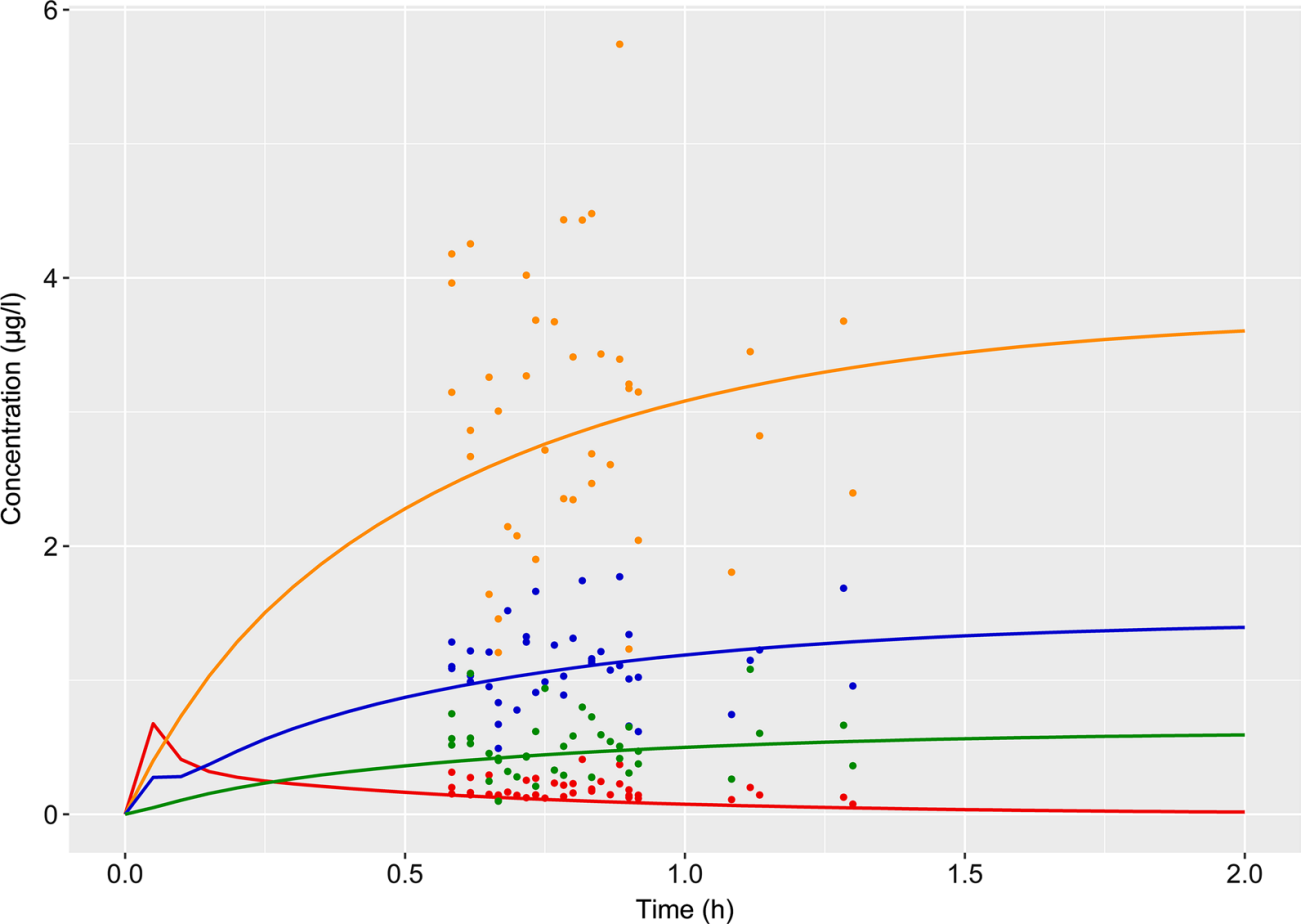
Simulation curves (lines) and observed clinical data (points) of ^68^Ga-DOTATATE over time with plasma concentrations in blood (red) and organ concentrations for spleen (orange), liver (blue) and thyroid (green)

Simulated concentration time curves for ^68^Ga-DOTATATE blood and spleen, liver and thyroid concentrations are depicted in Fig. 2. The SSTR2 concentration in the interstitial compartment of the spleen was estimated 112.0 nmol/L (total amount of 4.40 nmol in spleen) based on observed ^68^Ga-DOTATATE concentrations. For liver and thyroid, interstitial SSTR2 concentrations of 20.2 nmol/L (total amount 7.80 nmol) and 4.71 nmol/L (total amount 0.0108 nmol) were estimated, respectively. The renal plasma clearance was scaled to 0.67 ml/min/kg, resulting in a 12% unchanged excretion in the urine within 4 h. The total error of the model was 1.80, and results of the sensitivity analysis are shown in Table 3. Sensitivity values were satisfying and showed that the model was not highly reliant on particular input values.

**Table 3:** Sensitivity analysis results for the PK output parameter area under the concentration–time curve. Only sensitivity values < −0.5 or > 0.5 were reported

Compartment	PK output parameter	Input parameter	Sensitivity value
Spleen	AUC	^68^Ga-DOTATATE dose	1.03
Spleen	AUC	Fraction vascular of spleen	0.53
Liver	AUC	^68^Ga-DOTATATE dose	1.02
Liver	AUC	Liver volume	− 0.61
Thyroid	AUC	Thyroid volume	− 0.96
Thyroid	AUC	^68^Ga-DOTATATE dose	0.96

AUC: area under the concentration–time curve (0–24 h)

**Fig. 3:**
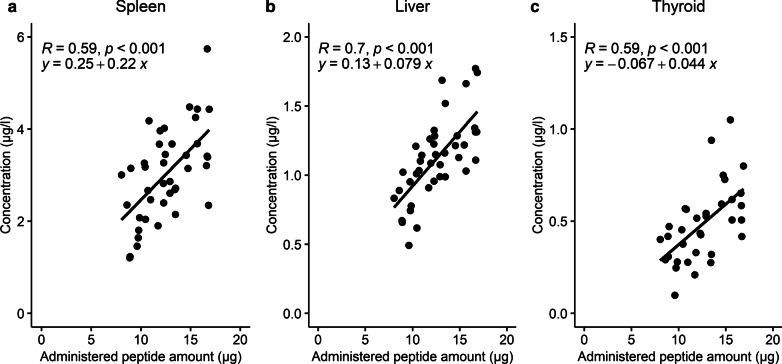
Observed peptide uptake (µg/l) for spleen (**a**), liver (**b**) and thyroid (**c**) versus administered DOTATATE amount (µg) for 41 patients without neuroendocrine tumors, where a correlation was observed that higher administered peptide amounts seem to result in increased uptake in these organs

**Fig. 4:**
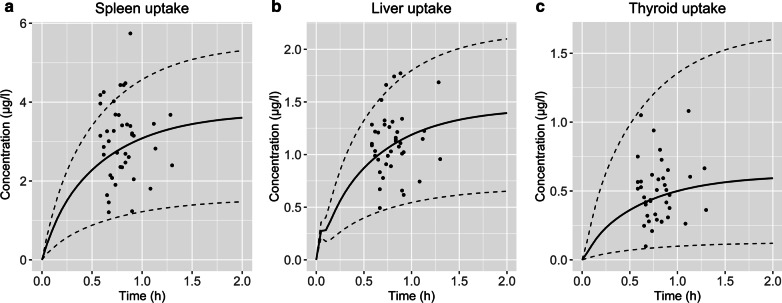
Predicted ^68^Ga-DOTATATE concentration–time curves (solid lines) for spleen (**a**), liver (**b**) and thyroid (**c**). Ranges (dashed lines) are based on minima and maxima of administered peptide amounts (8.05 and 16.9 µg) and SSTR2 density

The observed data showed evident variability for organ concentrations, especially for spleen. This was partly described taking into account the differences in administered peptide dose. For the clinical data in spleen, liver and thyroid, a correlation was noticed that higher administered peptide amounts resulted in a higher observed organ concentration (Fig. 3). Furthermore, varying other input parameters showed that SSTR2 density has a major impact on the total internalized amount of ^68^Ga-DOTATATE. Therefore, SSTR2 concentrations were manually fitted to a minimum and maximum based on the observed scan data for spleen, liver and thyroid. This resulted in fitted SSTR2amount ranges of 0.661–12.5 nmol, 3.04–11.3 nmol and 0.00281–0.0230 nmol for spleen, liver and thyroid, respectively. Using the administered peptide amount and SSTR2 density ranges as input for the simulation, 83%, 93% and 100% of all data points were within the predictions of concentrations for spleen, liver and thyroid, respectively (Fig. 4).

## Discussion

The aim of this study was to develop a PBPK model to describe ^68^Ga-DOTATATE organ distribution in patients without any detectable NETs, which can be used as starting point for further optimization of the use of this radiopharmaceutical. Evaluation and fitting of this PBPK model was performed using normal organ uptake in PET-data of 41 patients, which showed that the model could adequately describe these data. Furthermore, these predictions revealed the impact of peptide amount and inter-individual SSTR2 expression variability on the organ uptake of ^68^Ga-DOTATATE. These insights are useful for eventually predicting ^68^Ga-DOTATATE tumor distribution in patients with NETs and optimize diagnostic PET-imaging.

### Importance of peptide amount

The developed PBPK model showed the importance of administered peptide amount on ^68^Ga-DOTATATE uptake in normal organs. This phenomenon was previously described for tumors with high perfusion, but the range of administered peptide in this study was lower compared to the previous study of Kletting et al. (8–17 µg compared to 19–81 µg, respectively) [24]. In addition, findings regarding peptide amount were in agreement with several other PBPK models for SSA that also showed an effect of peptide amount on tumor and organ uptake [16, 47]. To retain a constant apparent specific activity of ^68^Ga-DOTATATE after labeling, the peptide amount used for labeling should be adjusted according to the ^68^Germaniun (^68^Ge) activity in the ^68^Ge/^68^Ga generator. However, at most in-house production sites a fixed peptide amount is combined with the generator eluate. This, combined with the inter- and intra-generator variation in ^68^Ga yield [48], explains the variability in administered peptide amounts. If the relationship between peptide amount and tissue accumulation could be demonstrated in a prospective clinical setting, then it would have major consequences for quantification of uptake on PET/CT and hence ^68^Ga-DOTATATE response prediction. Addition of extra information to this model (e.g., injected activity and absorbed doses) is rather easily performed, providing that this initial developed PBPK model could serve as a tool to identify appropriate peptide dose levels for such a trial, and thus limiting the need for a trial-design with various patient cohorts to assess many different dose levels.

Furthermore, it is important to recall that concentrations were calculated based on decay-corrected amounts at preparation time, where an excess peptide was labeled to a specific activity (i.e., an unknown amount labeled versus unlabeled DOTATATE was administered to patients). As the peptide uptake in organs was based on the activity concentration measured using PET data, an equal in vivo peptide distribution for labeled and unlabeled peptide was assumed. Therefore, it was also required to make the assumption that no receptor saturation or binding competition occurs within the range of total peptide amounts administered. This is a reasonable assumption, since no saturation processes have been described for somatostatin analogues using these low amounts of radiolabeled somatostatin analogues [16, 49, 50]. Moreover, our data showed a linear correlation between administered peptide amount and organ uptake observed at the PET-scan, indicating no saturation within the range of administered peptides (Fig. 3).

### SSTR2 density

With the developed PBPK model, a better understanding of physiological SSTR2 density in three organs was obtained, with the advantage that a large population was used to validate the output parameters such as this SSTR2 density. Estimation of SSTR2 densities was challenging using these low administered peptides amounts, because of the lack of information regarding receptor saturation. However, all other input parameters that might affect ^68^Ga-DOTATATE uptake were based on literature values, and it was assumed that SSTR2 density was the only parameter that could clearly explain differences between initial predictions and observed concentrations. Although this rationale supports the possibility that estimated SSTR2 densities approach correct values, it remains important to compare optimized values to previously reported SSTR2 densities. The estimated SSTR2 concentration in the spleen of 112.0 nmol/L seemed to be higher as reported by other PBPK models [16, 17]. However, it should be noted that the volume of this prediction refers to the interstitial compartment of the spleen (0.031 L), since SSTR2 is expressed on cell membranes. The total amount of SSTR2 in spleen (4.40 nmol) is comparable to these published in PBPK models by Maaß et al. and Kletting et al. [16, 17]. The results of the liver having a lower SSTR2 density than spleen are also in agreement with the literature, although this relative difference (20.2 nmol/L compared to 112.0 nmol/L) was even larger than a previously reported fivefold by Boy et al. [51]. This larger difference was also observed in a previously published PBPK model regarding uptake of ^90^Y-DOTATATE, although Kletting et al. reported a lower total amount of SSTR2 in liver compared to our predictions [16]. SSTR2 amounts were not fitted to observed data for kidney, because PET scan data were not suitable for whole compartment predictions. VOIs placed within the kidney resulted in activity that was mainly located intracellularly or within urine, while distinction between both locations was not possible. However, the SSTR2 amount in kidney was 2.95 nmol based on the initial input fraction and this corresponded to amounts published in other PBPK models [15–17]. Large variability in ^68^Ga-DOTATATE uptake in spleen was also reported by Sandström et al. and Walker et al. [8, 9]. Based on this PBPK model, this high variability in spleen uptake is probably due to a combination of actual inter-individual variability in SSTR2 density and administered peptide amount. Although not completely described by this PBPK model, the variability in ^68^Ga-DOTATATE spleen uptake did correspond to what is visually observed on scans in clinical practice.

### Blood observations

Results from the PBPK simulations in Fig. 2 showed that observed blood concentrations were slightly underestimated by the model. This underprediction is a result of optimizing the model fit only based on organ observations. Still, this organ-based PBPK model seemed to describe ^68^Ga-DOTATATE whole-body distribution best and was maintained for multiple reasons. Firstly, aorta data derived from PET-scans are more affected by the partial volume effect and noise than organ measurements due to the relatively small VOIs and low signal intensity. The fact that noise and methodology affect these measurements has also been demonstrated previously, as the average SUV_max_ for aorta in the current study was almost double compared to the reported values [42, 52]. This difference could well be explained by the method of defining the blood pool; in the current study, a circular VOI was placed within the aortic arch, while in the other study a less specific large VOI was placed over the mediastinum, thus including different tissue types with low ^68^Ga accumulation. Secondly, although blood predictions were lower compared to data observations, the predicted rapid plasma clearance showed a strong decrease after 45 min (Fig. 2), which was in accordance with the previous literature [53]. Lastly, estimation of the SSTR2 density using both aorta and organ observations resulted in worse distribution predictions. Then, an almost fourfold lower value was estimated for SSTR2 concentrations in spleen, which was not in line with previous findings [15–17]. In addition, the plateau of accumulation was then reached at ~ 3 h after administration, while in the current PBPK model the plateau of ^68^Ga-DOTATATE accumulation in organs occurs at ~ 1 to 2 h, which is in agreement with clinical reports [53]. To summarize, since the aim of this study was the development of a PBPK model describing ^68^Ga-DOTATATE whole-body distribution best, aorta observations were considered less important regarding SSTR2 reference concentration parameter fitting and these observations were exclusively used for visual model validation.

### ***Translation from ***^68^***Ga- to ***^***177***^***Lu-DOTATATE***

Despite the fact that the ^68^Ga- and ^177^Lu-DOTATATE were introduced as identical twins to serve the theranostic approach, there are important differences between the two that hamper direct translation of the ^68^Ga-DOTATATE PBPK model. The plasma protein binding of ^68^Ga-DOTATATE is, for instance, lower (31%) compared to ^177^Lu-DOTATATE (ca. 50%) [29, 42, 54]. This difference could be attributed to the higher lipophilicity of ^177^Lu-DOTATATE [41] or to the specific radioisotope, which may have an effect on the binding to plasma proteins. It is important to understand these radiopharmaceutical characteristics, because our sensitivity analysis indicated that fraction unbound had a high impact on the blood and organ distribution predictions. Also, *K*_D_ and blood flow have a relevant impact on peptide distribution [24, 47]. Another major difference between the ^68^Ga- and ^177^Lu-DOTATATE products is the amount of peptide that is administered (typically 20–50 µg versus 250 µg, respectively). Although these characteristics can be taken into account in a PBPK model, they do have a major impact on tissue distribution and tumor targeting [16, 24, 47]. Adjusting these input parameters to values specific for ^177^Lu-DOTATATE would make it possible to efficiently predict ^177^Lu-DOTATATE distribution as well. An example of such an approach is a previously developed PBPK model for PSMA-specific ligands [55].

### Limitations of PET-based PBPK modeling

A challenge in using PBPK for the prediction of kinetics of radiopharmaceuticals is the window of PK sampling. Often, scan time (the “sampling” moment) is based on target-to-background ratios and activity at time of scanning. Therefore, when using clinical PET data, all sampling moments lie in a rather small window, unless dynamic scanning is used. In our collected data, all PET/CT scans were performed within 35 and 78 min after injections, but as this PBPK model was designed to describe biodistribution (which occurs very rapidly after injection), the need for data on later time points is less essential. Hence, the simulation part regarding the degradation and excretion of ^68^Ga-DOTATATE should be interpreted with caution.

Organ predictions were based on the assumption that all compartments related to that specific compartment contribute to ^68^Ga-DOTATATE concentrations observed on PET scans. This assumption seemed reasonable, since in PET-based modeling voxel sizes of ~ 2 to 4 mm^3^ are common, so distinction between intra- and extracellular accumulation is impossible. This shortcoming as well as the inability to distinguish between intact ^68^Ga-DOTATATE, its metabolites or even unbound ^68^Ga is inaccuracies inherent to nuclear imaging. These inaccuracies combined with the complexity of peptide targeting could hold a clue for the limited value of ^68^Ga-DOTATATE for prediction of ^177^Lu-DOTATATE accumulations.

## Conclusion

To conclude, a whole-body PBPK model was developed to predict tissue distribution of ^68^Ga-DOTATATE in patients without detectable NETs and this model was evaluated using patient scan data. The model predicted SSTR2 amounts in spleen, liver and thyroid of 4.40, 7.80 and 0.0108 nmol, respectively. The administered peptide amount is an important factor to take into account for biodistribution studies. Furthermore, inter-individual variability in SSTR2 density was high, explaining the large variations also observed when assessing ^68^Ga-DOTATATE PET/CT.

## Data Availability

The datasets analyzed during the current study are available from the corresponding author on reasonable request.
